# Beyond the Mind—Serum Trace Element Levels in Schizophrenic Patients: A Systematic Review

**DOI:** 10.3390/ijms21249566

**Published:** 2020-12-15

**Authors:** Jacek Baj, Alicja Forma, Ryszard Sitarz, Kaja Karakuła, Wojciech Flieger, Monika Sitarz, Cezary Grochowski, Ryszard Maciejewski, Hanna Karakula-Juchnowicz

**Affiliations:** 1 Department of Human Anatomy, Medical University of Lublin, 20-400 Lublin, Poland; maciejewski.r@gmail.com; 2 Chair and Department of Forensic Medicine, Medical University of Lublin, 20-090 Lublin, Poland; aforma@onet.pl; 3 Chair and 1st Department of Psychiatry, Psychotherapy and Early Intervention, Medical University of Lublin, Gluska Street 1, 20-439 Lublin, Poland; e.sitarz@hotmail.com (R.S.); kaja.karakula@gmail.com (K.K.); karakula.hanna@gmail.com (H.K.-J.); 4 Faculty of Medicine, Medical University of Lublin, Aleje Racławickie 1, 20-059 Lublin, Poland; wwoj24@wp.pl; 5 Department of Conservative Dentistry with Endodontics, Medical University of Lublin, 20-090 Lublin, Poland; mksitarz@gmail.com; 6 Laboratory of Virtual Man, Chair of Anatomy, Medical University of Lublin, 20-400 Lublin, Poland; cezary.grochowski@o2.pl; 7 Department of Clinical Neuropsychiatry, Medical University of Lublin, Gluska Street 1, 20-439 Lublin, Poland

**Keywords:** schizophrenia, trace element, serum, psychiatry

## Abstract

The alterations in serum trace element levels are common phenomena observed in patients with different psychiatric conditions such as schizophrenia, autism spectrum disorder, or major depressive disorder. The fluctuations in the trace element concentrations might act as potential diagnostic and prognostic biomarkers of many psychiatric and neurological disorders. This paper aimed to assess the alterations in serum trace element concentrations in patients with a diagnosed schizophrenia. The authors made a systematic review, extracting papers from the PubMed, Web of Science, and Scopus databases according to the Preferred Reporting Items for Systematic Reviews and Meta-Analyses (PRISMA) guidelines. Among 5009 articles identified through database searching, 59 of them were assessed for eligibility. Ultimately, 33 articles were included in the qualitative synthesis. This review includes the analysis of serum levels of the following trace elements: iron, nickel, molybdenum, phosphorus, lead, chromium, antimony, uranium, magnesium, aluminum, zinc, copper, selenium, calcium, and manganese. Currently, there is no consistency regarding serum trace element levels in schizophrenic patients. Thus, it cannot be considered as a reliable prognostic or diagnostic marker of schizophrenia. However, it can be assumed that altered concentrations of those elements are crucial regarding the onset and exaggeration of either psychotic or negative symptoms or cognitive dysfunctions.

## 1. Introduction

Schizophrenia is a multifactorial psychiatric condition with a complex pathophysiological theory. It is described as a disruption of thought processes and an inconsistency between thoughts, emotions, and behavior. Schizophrenia prevalence is about 0.7%, with an onset in the early 20s among males and approximately 3 to 4 years later among females; its incidence is also 1.15-fold higher for men [1,2]. Patients diagnosed with schizophrenia suffer from psychotic symptoms: hallucinations, delusions, and negative symptoms, which include social withdrawal or flat affect, as well as cognitive impairments [3,4,5]. As a multifaceted condition, schizophrenia has a wide spectrum of hypotheses, among which alterations and abnormalities within neurotransmission (dopamine hypothesis) seem to be of primary relevance [6,7,8,9,10,11]. Other hypotheses refer to genetic predisposition, altered γ-aminobutyric acid (GABA) levels, nicotinic receptors, the endocannabinoid system, inflammation with oxidative stress, sex and developmental differences in brain anatomy, the protective role of estrogens, gut microbiota, or vitamin D deficiency [12,13,14,15,16,17,18,19,20,21,22,23].

The onset of psychiatric conditions or exaggerations of their symptoms might be induced by numerous factors including genetic predispositions, infections, inflammations, trace elements and vitamin levels, severe stress, postpartum complications, intellectual disability, neurodegenerative diseases, trauma, stimulants, and socio-economic and psychological factors [24,25,26,27,28,29,30,31]. The importance of various trace elements’ concentrations in human organisms cannot be underestimated since they are a crucial factor responsible for either induction or exaggeration of numerous disease symptoms (Table 1) [32,33,34,35,36,37,38].

Furthermore, trace elements’ imbalances might constitute indicators of disease remission or progression. It is still debatable whether altered trace elements’ concentrations could be implicated in the increased probability of schizophrenia onset. Numerous trace elements are crucial in the maintenance of proper functioning within the central nervous system (CNS) and these include physiological ranges of chromium (Cr), Cu, Fe, Mn, Se, or Zn. Therefore, pathological alterations in trace elements’ levels might contribute to the adverse impairments of the biological processes, inducing various diseases of the CNS or exacerbate already existing symptoms of autism spectrum disorder (ASD), neurodegenerative disorders (Alzheimer, Parkinson, Huntington diseases), dementia, major depressive disorder, or attention deficit hyperactivity disorder (ADHD) [144,145,146,147,148,149,150]. Several studies have shown altered levels of serum trace elements’ concentrations, as well as their impaired distribution within CNS in schizophrenic patients. Among numerous triggering factors, nutritional deprivation significantly affects the levels of certain trace elements, inducing oxidative stress, irrational behavior, and impaired cognition [151]. Besides, an association between metal exposure with subsequent concentration imbalances and psychotic symptoms’ severity was stated [152].

## 2. Aim of the Review and Search Strategy

**Figure 1:**
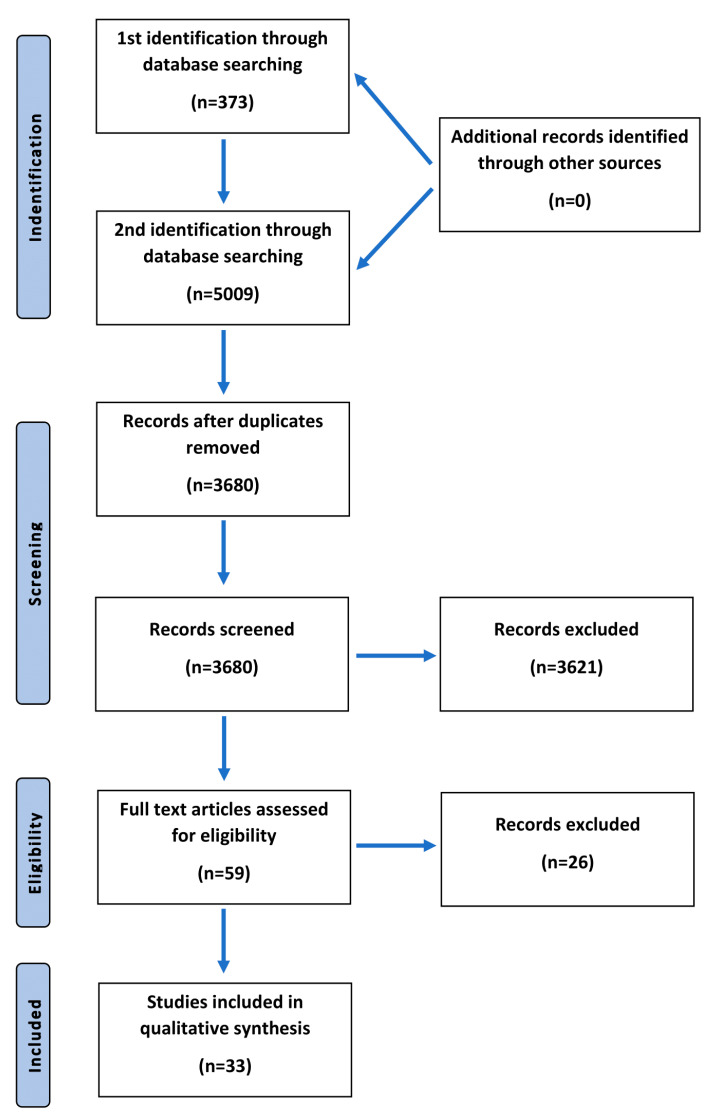
Flowchart presenting the process of article selection, according to Preferred Reporting Items for Systematic Review and Meta-Analyses (PRISMA) guidelines.

The objective of this paper was to conduct a review of the available literature regarding serum trace elements’ concentrations in schizophrenic patients. A systematic literature review of PubMed, Web of Science, and Scopus databases was performed by two identifications in March 2020. The first identification included the search strategy as follows: (schizophrenia OR schizophrenic) AND (trace element). After reviewing 373 articles searching for serum trace elements’ concentrations that had already been investigated in sera of schizophrenic patients, we chose the following ones to include in this review: magnesium, aluminum, zinc, copper, selenium, rubidium, potassium, cadmium, calcium, lithium, molybdenum, phosphorus, antimony, uranium, and manganese. We continued as the second identification with the use of the following search string: (schizophrenia OR schizophrenic) AND (magnesium OR aluminum OR cobalt OR zinc OR copper OR selenium OR rubidium OR potassium OR cadmium OR calcium OR lithium OR molybdenum OR phosphorus OR antimony OR uranium OR manganese). Eventually, 33 articles were included in a qualitative synthesis. The literature search included both human and animal studies. There were no restrictions regarding the year of a publication. The authors chose only articles in English. In the final analysis, regarding serum trace element concentrations in schizophrenics, we included only human studies. Twenty-six articles were excluded for several reasons: concentrations were measured in blood plasma, within erythrocytes, or in hair samples only; the aim of the study was not specifically focused on serum level of particular trace element; there were no sufficient, relevant, or accurate data; or it was investigated in a study, but lack of specific significant results disabled it from further analysis and description (Figure 1).

## 3. Results

After two identifications and duplicates were removed, we assessed 59 articles for eligibility, among which 33 of them were chosen in a qualitative synthesis. The articles included concerned the studies performed on humans. The time range of the published articles was 1950–2020. Two major inclusion criteria were the diagnosis of schizophrenia among patients and the examined trace elements’ concentrations in patients’ sera. Trace elements that were taken into consideration in this analysis included iron, nickel, molybdenum, phosphorus, lead, chromium, antimony, uranium, magnesium, aluminum, zinc, copper, selenium, calcium, and manganese. The results of the systematic review are included in Table 2.

## 4. Iron

Iron (Fe) metabolism is crucial regarding all neurobehavioral aspects since this element is involved in the metabolism of various neurotransmitters (serotonin, norepinephrine, dopamine), myelin synthesis, proper cellular functioning, and brain development [186,187,188,189]. The abovementioned neurotransmitters are synthesized by several Fe-dependent enzymes including phenylalanine hydroxylase, tryptophan hydroxylase, and tyrosine hydroxylase [190]. Therefore, Fe deficiency might decrease serotonin and dopamine levels, as well as increase urinary norepinephrine; this phenomenon is also associated with the direct involvement of Fe on hydroxyl radicals and reactive oxygen species’ formation [191]. Fe, being a required cofactor of cholesterol, is directly involved in the myelination processes [192]. Fe-mediated dopamine neurotoxicity is associated with quinones’ formation [193]. Animal studies of Fe concentrations showed an association between Fe deficiency, abnormal behavior, and alterations of dopamine metabolism [194,195]. Further, several human studies, as well as animal models of Fe depletion, presented significant behavioral and electrophysiological findings [196,197,198,199,200]. Fe deficiency is associated with developmental delays in children and cognitive impairments in adolescents; furthermore, it might affect motor development, recognition memory, socio-emotional behavior, and the overall processes of CNS maturation. Fe deficiency alters the density and activity of dopamine type 2 (D2) receptors by mimicking their blockade, which is implicated in the pathophysiology of schizophrenia [169]. Contrarily, increased Fe concentrations can lead to neuronal death primarily due to the enhanced oxidative stress, which might subsequently promote a progression of a wide range of neuropsychiatric disorders and neurodegenerative diseases [201,202,203,204,205]. Animal models have also shown that Fe overload might reduce dopamine levels in the striatum, induce neurodegeneration in the midbrain, and enhance the vulnerability to toxic injury [206]. Besides, increased Fe levels influence emotional behavior, which is determined by various biological and physiological indicators such as neurotransmitters’ concentration, density and affinity of neurotransmitter receptors, or Fe concentrations in specific brain regions [207]. Disturbed Fe metabolism impairs dopaminergic activity, resulting in a progression of negative symptoms of schizophrenia [169]. In the available literature, there is still no consensus on the altered levels of Fe in the serum of schizophrenic patients.

### 4.1. Serum Iron Levels

The results of the studies regarding serum Fe levels remain inconsistent. Generally, acutely schizophrenic patients present with decreased serum Fe concentrations [174]. Apart from lowered Fe levels, schizophrenic patients display decreased Mn and elevated Cu concentrations [208]. However, other researchers showed increased serum Fe and Ni levels among schizophrenic patients [156]. There is no correlation between Fe, Mn, Cu, Se, and Zn levels and duration of schizophrenia, doses of antipsychotic drugs, protein content, or smoking habits of patients. Despite altered serum Fe levels, there is no significant difference in ferroxidase II levels and Fe-binding capacity; besides schizophrenic patients present elevated levels of ceruloplasmin by approximately 20% [165]. Furthermore, schizophrenic patients exhibit a positive correlation between serum Fe concentrations and aspartate aminotransferase (AST) with alanine transaminase (ALT) levels [156]. It was suggested that altered serum Fe levels in schizophrenic patients might be due to the imbalanced diet, rather than being a separate characteristic of schizophrenia [181]. Moreover, increased Fe, Cu, Pb, Ca, Se, and Mn serum levels might be protective, whereas increased B, Cr, Mg, K, and As levels might constitute risk factors for schizophrenia [161]. Due to significant inconsistencies regarding serum Fe levels in schizophrenic patients, serum Fe levels can be considered neither as diagnostic nor as a prognostic factor of schizophrenia.

### 4.2. Iron Levels in the CNS

Casanova et al. observed a greater mean content of Fe per internal segment of globus pallidus in the post-mortem study of schizophrenic patients; however, these findings might be limited due to the small number of patients [209]. Even though several post-mortem studies showed the presence of basal ganglia mineralization (especially Fe deposition) in schizophrenic patients, the results remain inconsistent among researchers [210,211]. Microscopic examination of basal ganglia of schizophrenics revealed Fe deposition in the walls of small blood vessels [209]. Other studies showed no significant alterations in Fe, Cu, Zn, Mg, and Ca concentrations in the post-mortem brains of schizophrenic patients and control groups. However, both schizophrenic patients and control groups present with increased Fe and Cu concentrations in the caudate nucleus compared to the hippocampus and amygdala [212,213]. Demmel et al. presented a case of a schizophrenic patient whose element concentrations (Fe, Co, Rb, Se, and Zn) were at normal range in contrast to patients with alcohol abuse (decreased levels of Rb in cerebral nuclei and Co with Rb in cortical regions) or endogenous psychosis (increased levels of Fe, Co, Rb, Se, and Zn in the caudate nucleus) [214].

To the best of authors’ knowledge, there is no study that was investigating serum Fe and Fe levels in the brain at the same time. According to the reviewed literature, serum Fe levels tend to be decreased in schizophrenic patients. Contrarily, post-mortem studies focusing on Fe levels in the CNS showed that Fe might accumulate in several regions such as globus pallidus or basal ganglia.

### 4.3. Prenatal Iron Deficiency

Maternal Fe deficiency is an early environmental factor, which significantly enhances the probability of schizophrenia onset in offspring since Fe is involved in early behavioral and reflex neurodevelopment [169,186,215,216,217,218]. Moreover, low maternal hemoglobin concentrations might be associated with a nearly 4-fold elevated risk for schizophrenia in offspring [219]. The mechanism involves anemia induced by maternal Fe depletion and further reduction of oxygen delivery in the developing fetus [220]. Since proper Fe metabolism is a crucial component in myelination and dopaminergic transmission, any pathological alterations in its levels might disrupt fetal neurodevelopment. Apart from schizophrenia, fetal or postnatal Fe deficiency might influence the development of other psychiatric conditions including ASD, intellectual disability, hyperactivity disorder, anemia, anxiety, or depression in offspring [219,220,221].

### 4.4. Schizophrenic Patients with Akathisia

There is a significant inconsistency in the results of the studies regarding serum Fe levels in schizophrenic patients with coexisting symptoms such as akathisia. Barnes et al. showed that schizophrenic patients with chronic akathisia do not present lowered serum Fe concentrations; further, there is no relationship between serum Fe levels and the severity of akathisia [167]. Similar results were presented by Soni et al., where serum Fe, ferritin, and total iron-binding capacity (TIBC) parameters were not altered in schizophrenic patients with neuroleptic-induced akathisia [171]. Likewise, schizophrenic patients with acute dystonia are not characterized by low serum Fe, transferrin, or ferritin levels [172]. Wirshing et al. suggested that serum ferritin levels are associated with choreoathetoid movements especially among male schizophrenic patients treated permanently [173].

Contrarily, other studies showed that schizophrenic patients (either akathisic or non-akathisic) have significantly lowered serum Fe and ferritin levels compared to control groups [170]. The difference was more pronounced in schizophrenics with akathisia; those patients also presented elevated TIBC values. Furthermore, male schizophrenic patients present lowered ferritin but higher serum Fe levels comparing to females [168].

### 4.5. Serum Iron Levels during Treatment

The majority of studies have proven that antipsychotic treatment induces alterations in trace elements serum concentrations in schizophrenic patients, among which Na, Ca, K, Mg, Al, P, Zn, and Fe are mostly affected. However, several studies proved that Fe, ferritin, or transferrin levels may remain unchanged during treatment with neuroleptics [172]. Sussulini et al. (2017) observed that antipsychotic treatment (risperidone, olanzapine, quetiapine) induces the increase of Fe levels only in good responders, which provides the possibility to investigate Fe levels in monitoring responses to antipsychotic treatment among schizophrenic patients [222]. Contrarily, Chen et al. showed that antipsychotic drugs decrease Fe and increase P serum levels [157]. A long-term haloperidol treatment leads to Fe deficiency anemia, possibly due to haloperidol chelation abilities [166]. Furthermore, haloperidol-induced Fe deficiency alters the dopamine receptor D2 (DRD2) number. The reduction of serum Fe and ferritin levels with physiological TIBC status might be due to lurasidone treatment [223]. Besides, Li treatment significantly increases serum Fe concentrations in schizophrenics when compared to healthy controls [154].

## 5. Serum Nickel, Molybdenum, Phosphorus, Lead, Chromium, Antimony, and Uranium Levels

Increased Ni levels might induce alterations within the central as well as peripheral nervous systems, proving its cytotoxicity by various underlying mechanisms including mitochondrial dysfunctions [224]. Accumulation of Ni within the CNS disrupts neurotransmission and leads to the apoptosis of olfactory sensory and cerebral cortex neurons [191]. Further, some animal research showed that Ni exposure might increase the probability of aggressive behavior and affective disorders [225]. Ni affects the acetylcholine release, as well as decreases dopamine, norepinephrine, and serotonin levels within the CNS; it might also affect the gene expression of Glu receptors [226]. Nevertheless, literature specifically concerned with serum Ni levels in schizophrenic patients is scarce. Cao et al. (2019) showed that compared to healthy controls, patients with recurrent schizophrenic episodes presented elevated serum Ni and Fe concentrations [156]. Furthermore, an adjusted odds ratio (OD) revealed that serum Ni and Co levels are positively associated with schizophrenia.

Liu et al. did not point to any correlation between serum Mo or Zn levels and increased risk of schizophrenia [153]. However, patients with schizophrenia tend to present significantly lower serum concentrations of Mo [156].

Several studies suggest that altered P metabolism might reflect the degree of psychiatric symptoms and correlate with the presence of positive or negative symptoms [227,228]. Chen et al. observed that lower P levels are associated with increased schizophrenia risk and antipsychotic treatment might increase its serum levels, presenting its impact on the P metabolism [157]. These results were supported by Jamilian et al. who reported significantly lower levels of serum P in schizophrenics compared to healthy controls [181].

Ma et al. showed that increased serum antimony (Sb) and U levels might be associated with an elevated risk of schizophrenia [184]. Further studies showed that also increased serum concentrations of Pb and Cr constitute a risk factor for schizophrenia [185]. Even though, more research should be carried out in order to assess whether any of the above-described elements could act as a potential diagnostic and/or prognostic factor of schizophrenia.

## 6. Magnesium

Mg is the fourth most abundant element in human organism, involved in more than 300 enzymatic reactions including protein, DNA, and RNA synthesis [68]. Several human and animal molecular studies have proven its neuroprotective role. Its protective role in the nervous system includes the prevention of excessive excitation and potential excitotoxicity involved in the onset of numerous neurological disorders [71]. Therefore, any imbalances in Mg concentrations (primarily hypomagnesemia) might be associated with various disorders, including those with psychiatric/psychotic background such ADHD, depression, anxiety, Alzheimer and Parkinson diseases, migraine pain, epilepsy, or schizophrenia [229,230]. However, higher Mg and lower Zn levels are related to more severe psychopathology after metal exposure [152]. There is no consistency regarding serum Mg levels in schizophrenic patients [178,231,232]. However, it might be probably due to researchers’ tendency to determine only extracellular concentrations, while Mg constitutes an intracellular ion [233]. Furthermore, there are still no data regarding the concentration of ionized Mg levels in schizophrenic patients. Likewise, no altered levels in brain Mg concentrations in post-mortem studies have been reported [212]. Nevertheless, there is a significant number of studies regarding altered Mg levels after different types of medications used to treat schizophrenia.

### Serum Magnesium Levels during Treatment of Schizophrenia

Pimozide and fluphenazine induced a decrease of both serum Mg and Ca levels in the study by Alexander et al. on schizophrenics treated with neuroleptics [175]. Further studies confirmed the fact that treatment with pimozide, fluphenazine, or loxapine might slightly alter both serum Mg and Ca levels, also inducing extrapyramidal symptoms; however, according to researchers, the slight increase of Mg levels is not statistically significant [177]. Schizophrenic patients who tolerate higher doses of neuroleptics present higher serum Mg and Ca levels. Besides, Mg and Ca concentrations together correlate with pimozide dosage at which extrapyramidal symptoms might develop; this might be a result of impaired dopaminergic and cholinergic activity affected by altered Mg and Ca levels [176]. Contrarily, patients treated with major tranquilizers did not show any differences in serum Mg levels [178]. Mg levels in schizophrenics might be higher compared to healthy control groups, however, antipsychotic treatment (risperidone, clozapine, olanzapine, aripiprazole, quetiapine, perphenazine, sulpiride, ziprasidone) leads to a decrease of serum Mg levels [157,161].

## 7. Aluminum

Al, as a toxic metal, can accumulate in every organ particularly within the CNS, which is implicated in the onset or progression of neurodegenerative disorders. Increased Al concentrations are associated with the induction of oxidative stress, modification of membrane biophysics, impaired cell signaling, and neurotransmission [234]. Al increases the permeability of the blood–brain barrier and accumulation of this metal might induce the impairments of higher cognitive functions [235,236]. Besides, chronic Al exposure is associated with neurochemical, behavioral, and neuropathological impairments.

Kaya et al. observed higher Al, Fe, Cu, and Mg levels and lower Zn and Mg levels in the sera of schizophrenic patients compared to healthy controls [237]. Nevertheless, a more recent study by Liu et al. (2015) showed that lower concentrations of Al, Fe, Cu, Se, and As along with higher concentrations of Mg and Cr are associated with the increased risk of schizophrenia onset; however, these results were not statistically significant [153]. This inconsistency should be further evaluated in order to provide statistically significant results.

## 8. Zinc

Zn constitutes an essential trace element across the human lifespan, being involved in the control of early neonatal brain development and proper functioning of the CNS. Zn-containing neurons are found in the highest amounts primarily in the forebrain, being responsible for the interconnections between the limbic system and the majority of cerebral cortices [82]. At the molecular level, Zn is responsible for neuronal metabolism and plasticity, as well as synaptic activity. Thus, imbalances in Zn levels (especially increased concentrations) and further Zn accumulation within the CNS leads to neuronal damage via excitotoxicity, oxidative stress, and impaired cellular energy generation [80,238,239]. Zn deficiency during pregnancy and its insufficient levels during child development are associated with a higher probability of mental retardation, apathy, lethargy, and impaired learning ability [240,241]. Imbalanced Zn homeostasis is associated with an induction of various neurological and psychiatric conditions such as Alzheimer and Parkinson diseases, depression, amyotrophic lateral sclerosis, epilepsy, or schizophrenia [242,243,244].

### 8.1. Serum Zinc Levels

There is an inconsistency in the current literature regarding serum Zn levels in schizophrenic patients; nonetheless, in a majority of cases, serum Zn levels are usually reported to be decreased [183,245]. Cruz et al. showed significantly lower serum Zn, Se, and Fe levels, as well as higher Cu/Zn ratio among patients with schizophrenia compared to healthy controls [154]. Furthermore, the authors have suggested that Cu/Zn ratio might constitute a useful parameter related to oxidative stress in schizophrenics. Other researchers have suggested that there is no association between serum Zn and Mo levels and the increased risk of schizophrenia [153]. These results were confirmed by Cao et al., who showed that serum Zn, Mn, and Mo levels are negatively correlated, whereas serum Ni and Co levels are positively correlated with schizophrenia [156]. Besides, Saghazadeh et al. showed that schizophrenic patients tend to present lower Zn, Fe, and Mn serum concentrations, along with excessive Cu amounts [246]. Based on the results of the evaluated studies it can be assumed that Zn levels are rather decreased in schizophrenic patients; however, it cannot yet be stated whether serum Zn concentrations can be treated as either a prognostic or diagnostic factor of schizophrenia.

### 8.2. Zinc Levels in the CNS

A post-mortem study, which included 10 brains obtained from the schizophrenic patients during the autopsy, revealed the ionic Zn staining especially within the hippocampal formation [247]. Terminal zones of the mossy fiber system within the dentate hilus and the CA3 region of the hippocampus presented the highest density of Zn staining. A lighter Zn staining was present within the outer and inner layers of the dentate gyrus molecular layer, as well as within the CA1 region of the hippocampus. Another study, which included 12 subjects, showed that Zn levels were highest in the hippocampus, with lower Zn concentrations within the amygdala and the caudate nucleus; however, there were no significant differences between Zn levels among schizophrenics and a control group [212]. The results of these studies are intriguing since serum Zn levels in schizophrenic patients tend to be rather decreased. However, there are no studies that were investigating serum Zn and Zn levels in the CNS of schizophrenic patients at the same time.

### 8.3. Serum Zinc Levels during Treatment

Antipsychotic treatment leads to a decrease of serum Zn levels regardless of the treatment duration. Chen et al. observed that serum of both Zn and Cu were lowered after the antipsychotic treatment (which included risperidone, clozapine, olanzapine, aripiprazole, quetiapine, perphenazine, sulpiride, and ziprasidone) immediately, as well as after three weeks of treatment duration [157]. The authors also showed a significant decrease in serum Zn levels after risperidone treatment, whereas there was no association between Zn levels and olanzapine treatment. Valproate treatment was also reported to stabilize decreased Zn and K levels [248].

## 9. Copper

Cu is an essential micronutrient required for numerous cellular functions such as cellular respiration, neurotransmitter synthesis, neuronal myelination, or proper maintenance of Fe metabolism [249]. Cu constitutes a crucial element during the synthesis of ceruloplasmin, zyklopen, hephaestin, and dopamine-β-monooxygenase, which is crucial in dopamine metabolism and dopaminergic activity. Since Cu participates in reactions that generate free radicals’ contribution to oxidative stress, excessively high amounts of this transition metal might be toxic. Cu distribution within the brain parenchyma is generally even. However, the subventricular zone of brain ventricles was observed with higher Cu levels compared to other regions in the brain [250]. Post-mortem studies of Cu concentration within the CNS showed increased Cu concentrations mainly in the caudate nucleus in schizophrenic patients [212]. Disturbed distribution and homeostasis of Cu within the CNS contributes to the promotion of many neurodegenerative diseases: Alzheimer and Parkinson diseases, Huntington disease, amyotrophic lateral sclerosis, as well as psychiatric conditions such as depressive disorder, ASD, epilepsy, or schizophrenia primarily due to the impaired neuronal myelination, catecholamine imbalances, and disturbed brain architecture [251,252,253,254,255]. Neurobehavioral abnormalities might be a consequence of maternal Cu deficiency as well [186]. Schoonover et al. showed impaired intracellular Cu binding and transport into the cell via high-affinity Cu uptake protein 1 (Ctr1) in schizophrenic patients [256].

### 9.1. Serum Copper Levels

Regarding serum Cu concentrations among patients diagnosed with schizophrenia, there is still no consensus, as in the case of serum Fe and Zn levels. Several studies did not show any significant difference in serum Cu or ceruloplasmin levels in schizophrenics [159,160,162,163]. Other results have shown increased serum Cu levels among schizophrenic males and females compared to the control group [164]. Besides, serum Cu levels were higher in females, however, without statistical significance. Further, Cruz et al. observed increased serum Cu levels among patients with schizophrenia or bipolar disorder compared to control groups; however, these results were not statistically significant [154]. Cao et al. did not observe any significant differences in serum Cu, Co, and Fe levels in sera of schizophrenics [156]. A study on 40 male schizophrenic patients without any treatment for four weeks before the study revealed higher Cu, ceruloplasmin, LDL-cholesterol, total cholesterol, and high-sensitivity C-reactive protein (hs-CRP) levels, as well as a significant decrease in HDL-level [158]. Further, there was a significant correlation between serum Cu and hs-CRP levels. Wolf et al. also proved significantly elevated serum Cu levels among schizophrenics [165]. Furthermore, as approximately 95% of blood Cu is bound to ceruloplasmin, the researchers confirmed that increased Cu levels correlate with increased ceruloplasmin levels. Therefore, it can be assumed that schizophrenic patients tend to present greater serum Cu concentrations compared to healthy controls.

### 9.2. Serum Copper Levels during Treatment

Herran et al. showed that schizophrenic patients treated with a depot neuroleptic tend to have higher serum Cu levels compared to patients treated with oral antipsychotics [155]. The researchers observed that either typical or atypical antipsychotic treatment or neuroleptic dosage does not affect serum Cu levels. However, Saghazadeh et al. showed that schizophrenic patients treated with antipsychotic drugs tend to present higher serum Cu levels compared to controls [246]. Clozapine and aripiprazole treatment significantly decrease serum Cu levels compared to levels before treatment [157]. Intake of Se supplements elevates serum Cu and Zn levels [161].

## 10. Selenium

Various antioxidant selenoproteins such as glutathione peroxidase (GPx), thioredoxin reductase (TrxR), or selenoproteins—P, S, or H ((SelP), (SelS), or (SelH)) require incorporation of Se. Selenoproteins are involved in the free radical defense system, due to their antioxidant activity. They are responsible for proper CNS functioning. Furthermore, Se provides the response to oxidative stress primarily via mitochondrial biogenesis and regulation of Ca^2+^ channels [257]. SelP, being found primarily in neurons, also exhibits a neuroprotective effect. A neuroprotective role of Se supplementation is achieved via several mechanisms including antioxidant activity and selenoprotein synthesis de novo. Deficient or excessive Se exposure might be associated with the onset and progression of various diseases; however, the knowledge concerning the amount of exposure, as well as Se ranges and specific health outcomes, is still scarce [258]. Animal studies proved that Se deficiency induces neurological dysfunctions, examples of which are seizures, or neurodegeneration especially within the brain areas related to auditory and motor functions [259,260]. Indeed, patients with deficient Se supplementation or mutations in the gene encoding selenocysteine synthase are more susceptible to neurological impairments [261]. Imbalances in Se levels, as well as their disturbed distribution within CNS, might be a trigger of such conditions as schizophrenia, depression, bipolar disorder, or Alzheimer disease [100,149,262,263]. Schizophrenia and bipolar disorder are characterized by an elevated level of selenium binding protein 1 (SELENBP1) gene expression; however, it is yet unclear whether enhanced expression of SELENBP1 correlates with a higher incidence of psychosis [264,265]. It was hypothesized that schizophrenia onset might be not due to the Se deficiency itself, but rather due to the impaired Se transport [266,267].

### Serum Selenium Levels

Alertsen et al. did not show a statistically significant difference in serum Se levels between schizophrenics and the healthy controls [179]. A study on 114 schizophrenic patients revealed decreased levels of serum Se; the researchers also observed that lower serum Se, Fe, Cu, Al, and As might be associated with the increased risk of schizophrenia onset [153]. Schizophrenic patients usually present with reduced concentrations of serum Se [154,161,183]. Li et al. showed that Se supplementation by schizophrenics might increase appetite, as well as improve memory abilities—these results concern 60% and 18% of studied females and males, respectively [161]. Besides, the researchers observed that higher concentrations of Se, Mn, Ca, Pb, Fe, and Cu act protectively regarding schizophrenic patients. Thus, serum Se levels seem to be rather decreased in schizophrenics and the elevation of its concentrations might potentially improve the course of schizophrenia.

## 11. Calcium

Under physiological conditions, serum Ca levels and brain interstitial fluids maintain equilibrium; therefore, any changes in physiological Ca concentrations might affect neuronal and cognitive functions. Free intracellular Ca concentration in neurons is fairly low (a range of 10–100 nM), but some of the organelles such as mitochondria act as ‘Ca stores’ [268]. Numerous neuronal processes such as neurotransmitter release, intracellular signaling, proper homeostasis of glial cells, or N-methyl-D-aspartate (NMDA)-mediated neuroplasticity require the influx of extracellular Ca and its balanced homeostasis. Sharma et al. reported a relationship between higher Ca levels and poorer cognitive functioning in individuals aged 75 and above [269]. Recently, Ca^2+^/cAMP interaction, the so-called ‘calcium paradox’, was implemented to enhance neurotransmission and neuroprotection [270]. Further, voltage-gated Ca channels are implemented in the pathogenesis of schizophrenia. Other distortions associated with schizophrenia include altered C^2+^-dependent gene transcription, which affects signaling components. Schizophrenic patients present with increased Ca^2+^ intracellular levels, involving plasma membrane Ca^2+^ ATPase (PMCA) in the alterations of Ca levels [271]. The literature concerning serum Ca concentrations in schizophrenic patients is still scarce. Ruljancic et al. pointed to decreased serum Ca levels in suicidal schizophrenic patients compared to the nonsuicidal group of schizophrenics [180]. Besides, there is evidence that schizophrenic patients show an upregulation of Ca-binding proteins particularly within the cerebellum, which indicates impaired Ca signaling and disrupted cerebellar circuits [272]. Even though, more research should be conducted in order to assess whether any differences in serum Ca levels in schizophrenic patients are significant and whether they are truly associated with the course of this psychiatric condition.

## 12. Manganese

Studies suggest that some metals (Mn, Zn, Cd, Pb) are associated with schizophrenia or psychotic conditions [116,273]. These metals are involved in the induction of alterations in neurotransmission, excitotoxicity, and inflammation [112,274]. Excessive exposure to Mn might induce initial clinical psychotic manifestations, which include mood changes, emotional lability, hallucinations, and uncontrolled laughter, collectively referred to as ‘Mn psychosis’ [275]. Excessive Mn exposure is associated with behavioral and cognitive impairments, decreased IQ levels, reduced educational achievement, or abnormalities within the frontal lobe [276,277,278,279,280]. High levels of Mn were documented to cause Parkinson-like neuromuscular condition and neurocognitive dysfunctions that were primarily found in miners [274]. There is also evidence that large and long-lasting administration of manganese chloride (MnCl_2_) in rats, due to decreased activity of tyrosine hydroxylase, causes the development of schizophrenia-like behaviors [246]. It was also noted that higher concentrations of Mn decrease Gln uptake into astrocytes and reduce the activity of Gln synthetase in the neurons and astrocytes of the striatum and globus pallidus in the brain [281]. High level of free radicals due to the impaired activity of manganese superoxide dismutase (MnSOD) damage cell membranes causing malfunctioning of neurotransmission, abnormal neuronal apoptosis, and neurodegeneration, which is believed to induce the development of symptomatology in schizophrenia [282]. Researchers suggest that lower concentrations of Mn are associated with reduced MnSOD activity, which might be associated with schizophrenia [156]. So far, the majority of studies have shown that schizophrenic patients present with increased blood concentrations of Mn. However, Yanik et al. showed significantly lower plasma Mn concentrations in schizophrenics compared to control groups [208]. Besides, Cao et al. observed decreased serum Mn levels in cases of first-episode and recurrent schizophrenia, compared to healthy controls [156]. Contrarily, Saghazadeh et al. concluded that there are no significant changes in serum Mn concentrations in patients with schizophrenia; however, the researchers found lower levels of Mn in the subgroups of patients with previously diagnosed or chronic schizophrenia [246]. Plasma MnSOD activity is decreased in schizophrenic patients either with or without tardive dyskinesia [283]. Further, decreased MnSOD activity is associated with impaired cognitive functions in schizophrenics. Serum Mn levels might also be associated with an increased risk of schizophrenia [153]. Some studies suggest that Mn serum levels can be altered by psychoactive substances: They increase with the usage of tobacco or alcohol and decrease by antipsychotic medication [39,284,285]. Another evidence indicates that antipsychotic drugs do not alter the Mn concentrations but instead chelate Mn, making it less available as an enzyme activator [237]. Therefore, at the current state of knowledge, it is quite hard to assess what serum Mn levels might be associated with schizophrenia.

## 13. Conclusions

At the current state of knowledge, none of the investigated trace elements has presented its utility as a diagnostic or treatment tool of schizophrenia. The results of the analyzed studies were usually contradictory. Thus, the epidemiological evidence of altered serum trace element levels and the risk of schizophrenia onset is slightly controversial. The differences in the results of the abovementioned studies can be due to the heterogeneity of methodology, as well as different inclusion and exclusion criteria. Analytical methods regarding concentrations of trace elements are to some extent limited since, during gel electrophoresis or liquid chromatography, chelated metals can be easily released from proteins, preventing an accurate analysis [286]. Accuracy of the results might be to some extent limited particularly because the concentrations of trace elements are determined usually only in extracellular compartments, while some of the elements (e.g., Mg) are specifically intracellular ions; besides, for the majority of elements, there is a lack of strict concentrations’ ranges.

According to the results of the analyzed studies, maternal deficiency of essential trace elements with micro- and macronutrients along with subsequent dysregulation of early life trace elements’ concentrations might be associated with an increased risk of psychotic disorders including schizophrenia. Furthermore, the causation might be related to maternal stress, as it disrupts the placental transfer of nutrients. Prenatal deficiency of essential trace elements such as Zn induces epigenetic alterations, affecting the levels of other trace elements [152,287]. Alterations in serum trace elements’ levels in schizophrenic patients might be caused by numerous factors, among which current treatment (antipsychotics), exposure and inhalation of particular elements, or dietary intake are crucial.

Since the amount of literature is still scarce and the results are often contradictory, more research should be done in this field with the aim of investigating potential diagnostic and prognostic parameters of schizophrenia.

## Figures and Tables

**Table 1:** Physiological ranges and biological functions of trace elements in the human organism.

Element	Healthy Ranges		Biological Functions	References
	Male	Female		
Iron (Fe)	14–32 μmol/L (serum)	11–29 μmol/L (serum)	Fe is an essential trace element for all living organisms, as it constitutes a component of hemoglobin that is crucial in the storage and delivery of oxygen. Fe is required for the synthesis of hemoglobin, myoglobin, catalase, peroxidase, and cytochromes. Fe is a component of numerous proteins that are involved in DNA synthesis and cell proliferation. Fe homeostasis is crucial for proper mitochondrial functioning, cellular respiration, and subsequent ATP production; Fe is a component of several proteins involved in the electron transport chain. Fe is the most abundant trace element within the central nervous system (CNS), being involved in a vast number of processes including neurotransmitter synthesis (dopamine, serotonin), synaptic plasticity, and myelination. Proper Fe concentrations in the brain are regulated by ferritin. Thus, balanced Fe homeostasis and concentrations are crucial in the maintenance of proper cognitive functions and neurodevelopmental processes. Psychologically, Fe accumulation within the brain occurs in the elderly and might be associated with an induction of cognitive and motor dysfunctions. Any imbalances in Fe levels—either deficiency or overload—results in impaired monoamine neurotransmission, or cellular toxicity with neuronal damage respectively.	[39,40,41,42,43,44,45,46]
Molybdenum (Mo)	0.28 ng/mL–1.17 ng/mL (serum)		Mo is a cofactor for three main enzymes: sulphite oxidase (involved in the metabolism of sulfur-containing amino acids), xanthine oxidase/dehydrogenase (catalyzes oxidative hydroxylation of purines and pyridines), and aldehyde oxidase (oxidizes purines, pyrimidines, and pteridines). Sulphite oxidase deficiency might induce neurological symptoms. Low dietary Mo intake leads to decreased serum and urinary uric acid concentrations, as well as excessive xanthine excretion. Since Mo is required only in small amounts, its deficiency is infrequent. However, the so-called ‘acquired Mo deficiency’ is characterized by high blood methionine, low blood uric acid, and low levels of urinary sulfate and uric acid. Mo deficiency might be associated with motor dysfunctions.	[47,48,49,50,51,52]
Phosphorus (P)	2.5 to 4.5 mg/dL (blood)		P is an essential element for DNA and ATP synthesis, membrane synthesis, and protein phosphorylation. P constitutes a crucial component of DNA and RNA. Inorganic phosphate is crucial for proper skeletal mineralization—approximately 85% of P is distributed within bones and teeth, whereas the remaining quantities—in blood and other tissues. P is involved (either directly or indirectly) in the regulation of gene transcription, cell signaling (phosphorylation reactions regulation of acid-base homeostasis (maintenance of physiological pH of fluids), activation of numerous enzymes, or maintenance of proper storage of energy. P is also a component of 2,3-diphosphoglycerate. P deficiency is associated with numerous bone-related symptoms such as increased bone pain, fragility, or joint stiffness. Muscle dysfunctions are also very common and these mainly concern major muscles; severe cases might include respiratory depression and low cardiac output. Chronic P deficiency might result in proximal myopathy, rhabdomyolysis (risk of hemolytic anemia), and impaired erythrocyte synthesis. Hypophosphatemia is further characterized by neurological or cognitive symptoms such as fatigue, weakness, irritability, apathy, intention tremors, or even delirium, or coma. Contrarily, hyperphosphatemia manifests itself as enhanced vascular or soft tissue calcification, risk of secondary hyperparathyroidism, or renal osteodystrophy.	[53,54,55,56,57,58,59]
Nickel (Ni)	0.2 µg/L (serum)		Ni is highly abundant in nucleic acids, especially in RNA. Ni constitutes a component of several enzymes such as glyoxalase I, acireductone dioxygenase, nickel superoxide dismutase, ureases, Ni-Fe hydrogenase, methyl-CoM reductase, and CO dehydrogenase. Ni is involved in Fe absorption and erythrocytes synthesis, as well as adrenaline, glucose, hormones, lipids, and cell membranes metabolism. The most common side effects of excessive Ni exposure are lung fibrosis, skin allergies, and nasal, laryngeal, and lung cancers. So-called ‘Ni toxicity syndrome’ includes a vast number of symptoms including hypoglycemia, shortness of breath, nausea, lowered pulse, headache, diarrhea, vomiting. Severe Ni intoxication affects the respiratory tract and gastrointestinal system; the most common causes of death due to Ni intoxication are pneumonitis and cerebral edema.	[60,61,62,63,64]
Magnesium (Mg)	0.65–1.05 mmol/L (total Mg in serum) 0.55–0.75 mmol/L (ionized Mg in serum)		Approximately 99% of total body Mg in bones and muscles. Mg is a cofactor of more than 300 enzymes, which are involved in a vast number of functions including neuromuscular conduction, muscle contraction, myocardial contraction, or maintenance of blood pressure. Mg is required for glycolysis, energy production, and oxidative phosphorylation. It is involved in controlling N-methyl-D-aspartate (NMDA) receptors, preventing neuronal overstimulation. Mg is a crucial mineral for bone mineralization. It is essential for the maintenance of the structures of proteins, nucleic acids and mitochondria, and proper transmembrane transport of ions. Mg is also involved in several immunological functions such as macrophage activation, or lymphocyte proliferation. Mg deficiency might affect every system resulting in neurological (higher risk of migraine, stroke, seizure), gastrointestinal (insulin resistance, increased levels of triglycerides and total cholesterol), cardiovascular (increased risk of hypertension and atherosclerosis) symptoms. Such patients also present with a higher risk of osteoporosis. Hypermagnesemia manifests itself as hypotension, nausea, vomiting, cutaneous flushing, whereas more severe symptoms include neuromuscular dysfunctions, bradycardia, atrial fibrillation, respiratory depression, or a coma.	[65,66,67,68,69,70,71]
Aluminium (Al)	<10 µg/L (serum)		Patients who are exposed to high doses of Al are presented with Al accumulation in both blood plasma and brain. The entering of Al to the CNS is possible via transferrin and it mainly accumulates in those regions that are rich in transferrin receptors. Levels of Al, which might result in early symptoms of neurotoxicity are> 13 µg/L (plasma). Excessive Al exposure might induce inflammatory responses due to the increased expression of NF-κB and TNF-α. Cumulation of Al in the brain might induce several cognitive dysfunctions and in advanced stages—dementia. Further, it might impair hippocampal calcium (Ca) signaling pathways. Al neurotoxicity is associated with oxidative stress and impaired synthesis of acetylcholine due to the susceptibility of cholinergic neurons to Al; its excessive amounts also affect acetylcholinesterase (AChE) activity and impairs glial cells functions. Chronic Al exposure results in aluminosis, encephalopathy, breast cancer, or Alzheimer’s disease. Physiologically, Al content increases within the brain with age. Further, some research suggests that Al might be involved in chromatic compaction and epigenetics.	[72,73,74,75,76,77,78,79]
Zinc (Zn)	70–125 µg/dL (serum)		Zn is the second most abundant trace element in the human organism. Zn is involved in the maintenance of protein structure, regulation of gene expression, RNA, and DNA synthesis; thus, it is crucial in proper cell development, replication, and metabolism. Zn is a cofactor of numerous enzymes, including dopamine b-hydroxylase, monoamine oxidase, thyrosinase, alkaline phosphatase, carbonic anhydrase, superoxide dismutase, DNA and RNA polymerases, alcohol dehydrogenase, or matrix metalloproteinases. Zn is crucial for adult neurogenesis and proper hippocampal functioning. Zn inhibits the release of glutamate (Glu), as it inhibits γ-aminobutyric acid type A (GABAA) receptors. Within the CNS, Zn is most abundant in the hippocampus and the olfactory bulb, in the synaptic vesicles of glutaminergic neurons. Further, Zn-containing neurons are found within the forebrain in the highest amounts. At a presynaptic level, it can block Ca2+ channels, inhibiting neurotransmission. Zn-related alterations within the CNS can lead to cognitive impairments, mood disorders, anxiety, depression, epilepsy, Alzheimer’s disease, or dementia; generally, Zn is involved in the neuronal damage. Symptoms of Zn deficiency include growth retardation, mental lethargy, alterations in hormone metabolism, impaired immunity, and cognitive dysfunctions. Further, Zn deficiency is implemented in the promotion of inflammation.	[39,80,81,82,83,84]
Copper (Cu)	70–140 mcg/dL (blood)		Cu is the third most abundant transition element in the human organism and its highest concentrations are primarily found in the liver and brain. Cu is involved in proper Fe homeostasis, myelination, neurotransmitter synthesis, antioxidative defense, cellular proliferation, and signalling. Approximately 80–95% of plasma Cu is bound to ceruloplasmin; metallothionein constitutes a major protein for copper storage. It is a component of several enzymes such as tyrosine hydroxylase, dopamine hydroxylase (involved in dopamine and norepinephrine production), superoxide dismutase, and cytochrome c oxidase. Cu is capable to bind to GABAA, NMDA receptors, and voltage-gated Ca^2+^ channels, affecting synaptic transmission. Cu toxicity is primarily associated with free radical-induced oxidative damage. Cu is also crucial in the induction of inflammation, as it affects a lymphocytic release of interleukin-2 (IL-2). Imbalances in Cu concentrations and metabolism might be associated with neurodegenerative diseases such as Alzheimer’s disease, Menkes disease, Wilson’s disease, or spongiform encephalopathies. Further, Cu deficiency primarily results in B_12_ and folate deficiency, aplastic anemia, leucopenia, or myeloneuropathy.	[39,85,86,87,88,89,90,91,92,93,94]
Selenium (Se)	-		Se is incorporated in selenoproteins that are involved in antioxidant reactions (glutathione peroxidase (GPx), thioredoxin reductase (TrxR), iodothyronine deiodinases (IDD) enzymes), regulation of thyroid functions and thyroid hormone metabolism, or even immune protection. Proper development of GABAergic interneurons within the hippocampus and cerebral cortex depends on selenoproteins. Se also shows neuroprotection in the nigrostriatal pathway and maintains proper functioning of the dopamine pathway. Se is associated with the etiology with such neurological disorders as Alzheimer’s and Parkinson’s diseases, epilepsy, or amyotrophic lateral sclerosis. High concentrations of Se lead to toxicity of dopaminergic neurons. Severe Se deficiency might lead to such diseases as Keshan disease, Kashin-Beck disease, *myxedematous endemic cretinism*, as well as *i*mpairments of skeletal and cardiac muscles. Blood Se level over 100 μg/dL might be toxic and result in selenosis, which is manifested as fatigue, irritability, gastrointestinal dysfunctions, or neurological damages. High dietary Se intake might be related to a reduced risk of cancer.	[39,95,96,97,98,99,100]
Calcium (Ca)	8.6–10.2 mg/dL (blood)		Ca is the most abundant mineral in the human organism, which is found in 99% in bones and only in 1% in serum. Ca metabolism is strictly associated with numerous nutrients, among which P and vitamin D are ones of major importance. Ca is crucial in proper nerve transmission, vasoconstriction with vasodilation, muscle contraction, and intercellular signalling. Maintenance of proper Ca^2+^ homeostasis is provided by two types of Ca^2+^ transport ATPases—the plasma membrane Ca^2+^-ATPase (PMCA) and the intracellular sarco/endoplasmic reticulum Ca^2+^-ATPase. Ca sensing receptors are expressed by neuronal and glial cells and are activated by the extracellular Ca; these receptors are involved in neurotransmission and synaptic plasticity. Ca ions are involved in the initiation and regulation of responses to the injuries within the CNS. Astrocytic Ca signals can modulate synaptic transmission, further, proper glial cells function is controlled by Ca signalling. Ca overload might induce Glu excitotoxicity and stroke, as well as numerous neurodegenerative diseases. Ca deficiency induces calcification of cerebellum, cerebral cortex, and basal ganglia (with subsequent extrapyramidal signs), irritability, increased intracranial pressure, or spasms. Contrarily, hypercalcemia primarily results in endocrine disorders (due to increased parathormone production).	[101,102,103,104,105,106,107,108,109]
Manganese (Mn)	0.4–0.85 μg/L (serum)		Mn is a crucial element involved in the regulation of glucose and lipids metabolism, as well as synthesis and activation of many enzymes such as arginase, isocitrate dehydrogenase, phosphoenolpyruvate carboxykinase, manganese superoxide dismutase (MnSOD), glutamine synthetase, glycosyl transferases, pyruvate carboxylase. Thus, Mn is involved in proper development, antioxidant defense, energy production, immune responses, and neuronal activity. The highest concentrations of manganese within the CNS are primarily found in the putamen, caudate nucleus, and globus pallidus. Molecular mechanisms associated with Mn toxicity are numerous, among which oxidative stress, mitochondrial dysfunction, autophagy dysregulation, and apoptosis are one of the most important ones. Mn toxicity is associated with a disruption of the glutamine (Gln)/glutamate (Glu)- (GABA) cycle (GGC) between astrocytes and neurons impairing neurotransmission and Gln metabolism. Imbalances in Mn levels are implicated in neurodegenerative diseases. Excessive Mn levels (manganism) resemble Parkinson’s disease and include similar cognitive, motor, and emotional impairments; they might also induce encephalopathy. Excessive amounts of Mn are neurotoxic and the mechanism is enhanced by Mn-related overactivation of glial cells and further neuroinflammatory responses.	[39,110,111,112,113,114,115,116]
Lithium (Li)	0.14–8.6 μmol/L (serum)		Li is an alkali metal that except for human serum, it can be found in the erythrocytes, liver, and uterus. Li presents anti-manic properties and it is currently widely used in such psychiatric conditions as bipolar disorder, major depressive disorder, or mania. Similar to therapeutic Li, endogenous Li also has physiological functions in regulating mood. Lithium ions prevent the induction of the functional hypersensitivity to dopamine and acetylcholine receptors; it also increases the serotoninergic activities. Li affects neurotransmission by modulating Glu, dopamine, GABA, glycine, and acetylocholine transmission. Several studies showed positive effects of Li supplementation on the volume of gray matter and integrity of the white matter of the brain. Besides, Li is believed to increase the number of neutrophils and eosinophils as well as a proliferation of the T-cells. Li presents the ability to reserve the effects of oxidative stress.	[117,118,119,120,121,122,123,124]
Rubidium (Rb)	-		Rubidium is a metal belonging to the alkali metal group. Rubidium is considered to be the most abundant metal in the human organism that lacks any major biological function. Its half-life in the human organism is estimated to 31–46 days. Generally, this metal seems not to be toxic for humans, however, it is also easily absorbed. Rb ions are treated similarly to K ions and are stored in the body’s intracellular fluid. Since Rb ions resemble K ions, it is believed that those might present similar functions. However, the replacement of K ions with Rb ions (about 50%) was fatal, according to the results of the animal model. It was demonstrated that Rb might facilitate the turnover of the brain noradrenaline in rats and monkeys.	[125,126,127,128]
Potassium (K)	3.5–5.0 mmol/L (blood)		Proper K homeostasis is crucial in the maintenance of physiological, cellular functions. K is primarily an intracellular ion; only 2% of K ions are localized in the extracellular fluid. The physiological membrane potential is controlled by the Na^+^-K^+^ ATPase exchanger, which pumps Na^+^ outside the cell, replacing it with K^+^ ions. K ions are crucial for the maintenance of physiological fluid balance at the same time enabling proper nerve signaling and muscle contractions. Disturbed K levels are associated with the alterations of the blood pressure—greater K concentrations lead to lowered blood pressure, whereas low K levels induce hypertension. Na/K ratio is linearly associated with blood pressure. Besides, reduced K levels promote vascular calcification and aortic stiffness. Hypokalemia is much more common than hyperkalemia and is usually caused by chronic diarrhea, vomiting, or excessive loss of bodily fluids. Hyperkalemia is associated with muscle weakness and cardiac arrhythmias. K supplementation might be associated with a decreased risk of stroke; it also shows protective effects against several pathologies of kidneys (e.g., kidney stones) and bones.	[129,130,131]
Uranium (U)	-		U is a heavy metal that might be absorbed into the human organism via several routes including inhalation, ingestion (of U-contaminated food and water), and dermal contact (e.g., damaged tissues). U toxicity is either acute or chronic and the most vulnerable organs include the kidneys; other organs such as bones, liver, lungs, or reproductive organs might also be affected by chronic toxicity. Since U was showed to pass through the brain-blood barrier, it is also believed to have neurotoxic effects; so far U showed its toxic properties mostly towards dopaminergic cells. Besides, chronic U exposure may affect the immune system resulting in a wide spectrum of infectious diseases autoimmune diseases, or even induction of carcinogenesis.	[132,133,134,135,136]
Cadmium (Cd)	0.5–2.0 ng/mL (blood)		Cd is a toxic heavy metal of no major biological function in the human organism. Cd has been classified as a human carcinogen since it disrupts DNA repair enhancing uncontrolled cellular proliferation. Further, Cd facilitates the overexpression of numerous proto-oncogenes such as c-myc or c-jun [AE]. Cd also facilitates the induction of oxidative stress. Contamination with Cd is currently quite prevalent in most food products and Cd tends to accumulate in the human organism with age. Cd was observed to mostly accumulate in the liver, lungs, and eye tissues. Chronic Cd exposure might be associated with such diseases as Itai-itai disease or tubular impairments which is further linked to bone demineralization and osteoporosis. It was demonstrated that there is an association between Cd levels and the risk of diabetes, diabetic nephropathy, hypertension, or periodontal diseases. As a carcinogenic agent, chronic Cd exposure might induce tumorigenesis in various organs leading to lung, pancreatic, prostate, stomach, or bladder cancers. Moreover, chronic Cd exposure might lead to several neuropsychological dysfunctions including cognitive delay.	[137,138,139,140,141,142,143]

**Table 2:** The studies about serum trace element concentrations in schizophrenic patients included in the systematic review.

Ref.	Authors	Year	Origin	No. Patients	No. Controls	Age (SG)	Age (CG)	Sex	Trace Elements	Analytical Methods for Serum Trace Elements Detection	Results	Drugs/ Treatment	Additional Information
[153]	Liu et al.	2015	China	114	114	32.8 ± 11.3	33.0 ± 10.7	152 Females ^1^	Ni, Mo, As, Al, Cr, Mn, Se, Cu, Fe, Zn	Samples were determined by inductively coupled plasma-mass spectrometry (ICP-MS).	Ni, As, Al, Cr, Mn, Se, Cu, Fe are associated with the risk of schizophrenia (specifically: lower Ni, Cu, Se, As, and Al levels, as well as, higher concentrations of Cr and Mn) (*p* < 0.05), except for Zn and Mo. Further, the results showed that Cu ≤ 0.97 μg/mL, Se ≤ 72 ng/mL, and Mn > 3.95 ng/mL are associated with the risk of schizophrenia. SC present lower Se and Cu, as well as higher Mn levels compared to CG.	Patients were not treated with any mineral or vitamin supplements. ND about other treatment strategies.	All of the patients were diagnosed with schizophrenia. Patients with diabetes, kidney failure, or other coexisting diseases, as well as those with schizophrenia, but with additional psychiatric conditions—were excluded from the study.
								76 Males ^1^					
[154]	Santa Cruz et al.	2020	Brazil	11	11	33.7 ± 7.9	35.9 ± 7.0	3 (SG) 7 (CG) Females	Cu, Fe, Zn, Mg, Se, K, P, Ca	Elements concentrations were determined by the ICP-MS.	Schizophrenics present significantly lower serum Se and Zn concentrations compared to CG. Serum Fe levels are significantly higher (*p* < 0.05) in SC compared to CG. A significantly higher Cu/Zn ratio was determined in SG (ratio = 2.4, *p* = 0.028).	1 patient was treated with anticonvulsants, 2 with anxiolytics, 9 with antipsychotics, and 3 with antidepressants.	The whole studied cohort included 37 patients—11 schizophrenics, 11 controls, 7 with bipolar disorder treated with Li and 8 with bipolar disorder treated with medicaments other than Li. Control group included patients without any recognized psychiatric conditions. All of the patients did not have diabetes, kidney failure, or any other coexisting diseases.
								8 (SG) 4 (CG) Males					
[155]	Herrán et al.	2000	Spain	62	62	38.9 ± 11.7	38.0 ± 9.3	30 (SG) 30 (CG) Females	Cu, Zn	Cu and Zn concentrations were measured by atomic absorption spectrophotometry.	Cu levels were significantly higher (*p* = 0.004) in SG than in CG. There was a relationship within sex—females had greater serum Cu levels. Patients with neuroleptic treatment had higher Cu levels (mean 127.9 μg/dL; ±23.3), than those treated with oral antipsychotics (113.7 ± 22.8) (*p* = 0.036). Serum Cu levels were not affected by typical/atypical antipsychotics or current neuroleptic dosage. Zn levels were non-significantly higher in SG and male patients presented higher serum Zn levels. There were no differences in serum Zn levels in patients treated with neuroleptics.	Out of 62 patients, 5 were without pharmacological treatment. 17 patients were taking depot neuroleptics, and 19—atypical antipsychotics.	Patients with schizophrenia and other coexisting psychiatric conditions were excluded from the study. Additional exclusion criteria included: pregnancy, medical disorders (immune, endocrine, liver, cirrhosis), and drugs (which are known to affect trace elements levels). CG were not diagnosed with any psychiatric disorder, CG had never taken psychotropic drugs, or any kind of psychiatric treatment. None of the patients presented gastrointestinal disorders, or signs of malnutrition. Median of schizophrenia duration among SG is 11 years and 2 hospital admissions. SG primarily included patients with negative symptoms and residual schizophrenia.
								32 (SG) 32 (CG) Males					
[156]	Cao et al.	2019	China	105	106	29.3 ± 5.6	30.65 ± 4.6	61 (SG) 68 (CG) Females	Zn, Mn, Cu, Fe, Co, Ni, Mo	Elements concentrations were determined by the ICP-MS.	SG has reduced serum Mn (q < 0.001) and Mo (q = 0.009) concentrations, and increased Ni (q = 0.009) and Fe (q = 0.012) concentrations compared to CG. No significant changes were observed in cases of other trace element levels. First-episode schizophrenia characterized with lower Mn concentrations, whereas recurrent schizophrenia—with lowered Mn and Mo levels, compared to CG. Recurrent schizophrenia characterized with elevated serum Fe and Ni levels compared to CG.	SG was not taking any antipsychotic drugs for a minimum 1 month before hospitalization.	SG included patients who did not receive antipsychotic treatment for a minimum 1 month before hospitalization. HC was a group of patients without any current and/or past psychiatric conditions. Exclusion criteria were as following: <18 or >40 years old, exposure to heavy metal industry, coexisting diseases, presence of additional psychiatric conditions, current or recent pregnancy and/or breastfeeding. 24 of 105 patients from SG were drug-naïve and during first-episode schizophrenia, remaining 81 had recurrent and/or chronic schizophrenia. Additionally, several other paratemeters were studied: fasting blood glucose (FBG), triglycerides (TG), total cholesterol (TC), aspartate aminotransferase (AST), alanine aminotransferase (ALT), albumin (ALB), total protein (TP), creatinine (CREA), and uric acid (UA).
								44 (SG) 38 (CG) Males					
[157]	Chen et al.	2017	China	165	614	28.83 ± 12.54	37.45 ± 12.12	66 (SG) 518 (CG) Males	Mg, Cu, Ca, P, Fe, Zn	Serum Cu, Zn, and Fe were determined by the colorimetric method, serum Ca—by arsenazo III method, serum Mg—by xylidyl blue method, and serum P—by ultraviolet spectrophotometry method.	Serum Mg and P concentrations in SG were significantly higher compared to CG (*p* < 0.001). Serum Ca, Fe, and Zn were significantly lower—(*p* < 0.01), (*p* < 0.001), and (*p* < 0.001) respectively. Before treatment, serum Fe and Zn levels in SG, correlated negatively with–age—(*p* < 0.01) and (*p* < 0.05) respectively. In the whole population, CG and SG, Ca had a positive correlation with P, Fe, and Zn (all *p* < 0.05); Fe had a positive correlation with Zn (*p* < 0.05). In SG, serum Fe concentrations were significantly higher (*p* < 0.01) in males compared to females in both pre- and post- treatment groups. Serum Zn significantly decreased (*p* < 0.05), compared to the levels before treatment. After single drug treatment, serum P was significantly higher that P before treatment, whereas Zn levels decreased (both—*p* < 0.05). During a combined treatment, serum Cu and Zn decreased (*p* < 0.05), whereas P significantly increased (*p* < 0.001). Serum Cu levels were significantly lower after clozapine (*p* = 0.04) and aripiprazole (*p* = 0.02) treatment. After risperidone (*p* = 0.01), clozapine (*p* = 0.01), and aripiprazole (*p* = 0.003), serum P significantly increased. There was no association between olanzapine and serum trace element levels. After treatment, serum P increased significantly in mixed type schizophrenia (*p* < 0.001), paranoid schizophrenia (*p* <0.05), and acute schizophrenia (*p* < 0.05). Serum Zn levels decreased significantly in mixed schizophrenia (*p* < 0.01), acute schizophrenia (*p* < 0.05) and schizotypal schizophrenia (*p* < 0.05) compared to levels before treatment. Further, Fe levels were significantly higher in acute schizophrenic patients compared to schizotypal schizophrenia before treatment (*p* < 0.05).	Antipsychotic drugs included: risperidone, clozapine, olanzapine, aripiprazole, quetiapine, perphenazine, sulpiride, and ziprasidone.	Schizophrenic patients with cardiovascular/cerebral vascular disease, liver disease, nephropathy, and/or immunological diseases, as well as other concurrent diseases, were excluded from the study. SG was classified into four groups: mixed type SZ (*n* = 103), paranoid SZ (*n* = 21), acute SZ (*n* = 19), and schizotypal SZ (*n* = 15).
								99 (SG) 96 (CG) Females					
[158]	Devanarayanan et al.	2016	India	40	40	29 ± 6	27 ± 4	40 (SG) Males	Cu	Cu levels were measured applying reagent kits by micro-titer plate reader by colorimetric method.	Drug naive patients (*n* = 22) had significantly higher serum Cu levels compared to CG (*p* = 0.026). Serum Cu correlated significantly with high-sensitivity C-reactive High-sensitivity C-reactive Protein (hs-CRP) (*p* = 0.003). Serum Cu levels did not correlate with PANSS scores; serum Cu levels are not associated with the severity of schizophrenia.	SG was not taking any drugs for schizophrenia for 4 weeks before study.	SG constituted of 40 male patients (22 drug naïve and 18 drug free). Patients from SG included those who were not on any treatment for SZ for at least 4 weeks. Exclusion criteria were: concurrent psychiatric conditions, dependency on drugs (other than caffeine), unstable medical and/or neurological conditions. The same exclusion criteria were for CG. Despite serum Cu levels, blood glucose, total cholesterol, triaacylglycerol, high-density lipoprotein (HDL), very-low-density lipoprotein (VLDL), and low-density lipoprotein (LDL)-cholesterol, ceruloplasmin, and hs-C-reactive protein were investigated.
								40 (CG) Males					
[159]	Edelstein et al.	1959	Jerusalem	20	10	22–45 (range)	22–45 (range)	ND	Cu	Cu levels were determined by a photo-electric colorimeter (Lumetron model 402-E)	No significant increase in serum Cu in SG was found, compared to CG.	Patients did not receive any SZ treatment.	Among 20 schizophrenics, 10 were paranoid, 3 catatonics, 2 hebephrenics, 3 simplex, and 2 of the circular form.
[160]	Frohman et al.	1958	USA	16	11 (healthy) + 9 (with psychiatric conditions other than SZ)	ND	ND	ND	Cu, Fe	Bausch & Lomb spectrophotometer at 485 m/µ wavelength	CG and SG did not differ in serum cu levels. Serum Fe levels were lower in SG (*p* = 0.05), however serum Fe levels were lowered in all of the patients sera.		Among 16 patients, 11 had chronic SZ and 5 acute SZ. The duration of SZ was more than 2 years. In a CG: 3 patients were diagnosed as neurotic, 3 with manic-depressive disorder, 2 with personality disorder, and 1 with organic brain syndrome. SG and CG were on the same diet.
[161]	Li et al.	2017	China	158	669	45.46 ± 11.00	46.73 ± 12.58	72 (SG) Males	Cr, Zn, Se, Cd, Cu, Pb, As, K, Ca, Mg, Fe, B, Mn	Elements were detected by the ICP-MS.	Serum Mn, Se, Cd, Pb, Ca, Cu, and Fe in SG were lower compared to CG. Serum B, Cr, As, K, and Mg levels were higher in SG compared to CG. Serum Pb levels were the highest in the group <29 years old.	Therapy included risperidone, olanzapine, and quetiapine. 21 patients were taking Se supplements.	The duration of male SZ was between 1 and 45 years (mean 13 years). The course of female SZ was between 1 and 43 years (mean 12 years). 21 SZ patients (16 females, 5 males) with low Se levels were selected to take Se supplements. Daily dose of Se was about 60 μg. Serum was collected after 1 and 3 months after Se supplementation. CG did not have any concurrent diseases. Exclusion criteria for both groups (SG and CG) were: smoking and/or drinking, immune system disease, malnutrition, history of cardiovascular disease, trace elements supplementation.
								86 (SG) Females					
[162]	Maas et al.	1961	USA	20	20	ND	ND	ND	Cu	Sample incubation for 10 min with hydrochloric acid.	Serum Cu levels were not significantly different in SG compared to CG (patients with neuroses or personality disorder). There was also no significant difference between anxious and non-anxious groups.	ND	The whole study group included: 10 extremely anxious schizophrenics, 10 non-anxious schizophrenics, 10 extremely anxious nonpsychotic patients, and 10 non-anxious non-psychotic patients. Patients who have taken phenothiazines, antidepressants, or psychic energizers 2 weeks before, were excluded from the study. Patients with additional concurrent diseases were excluded.
[163]	Munch-Petersen	1950	Denmark	40	ND	21–50 (range) Males 23–48 (range) Females	ND	24 (SG) Males	Cu	Cu determination was done by the color reaction with sodium diethyldithiocarbamate.	Serum Cu levels in SG are within the physiological limits.	Most of the patients were treated with shock (by means of insulin, or combined with metrazol shock, or electroshock)	The duration of SZ in patients was between 2 and 32 years, and 4 and 24 years in males and females respectively.
								16 (SG) Females					
[164]	Olatunbosun et al.	1975	Nigeria	102	95	33 (SG) Males 32 (SG) Females	32 (CG) Males 31 (CG) Females	74 (SG) Males 28 (SG) Females	Cu	Cu levels were determined in deproteinized serum in a Beckman Model 495 Atomic Absorption Spectrophotometer.	The average serum Cu levels in SZ males was significantly higher compared to CG (*p* < 0.001). Likewise, serum Cu levels in SZ females was significantly higher compared to CG, however without any statistical significance.	Treatment of all patients was similar and included: chlorpromazine, amitriptyline, trifluoperazine, thioridazine, and electroconvulsive therapy.	The duration of SZ varied from 3 months to 15 years.
								67 (CG) Males 44 (CG) Females					
[165]	Wolf et al.	2006	USA	10	8	34.5 (mean age of SG + CG)	-	10 (SG) Males	Cu, Fe	In the serum samples, Fe was assayed by absorbance photometry by guanidine/ferrozine method. Cu was assayed by atomic absorption spectrophotometry.	SG had significantly higher serum Cu levels. Fe levels and iron binding capacity did not differ in both groups.	Three subjects did not receive any treatment, 7 were on ‘typical’ antipsychotic treatment. None of the subjects received typical antipsychotics.	All of the subjects met RDC broad criteria for schizophrenia. 8 subjects met DSM-III-R criteria for schizophrenia, 2 subjects—for schizoaffective disorder. All of the subjects did not have concurrent diseases, or history of substance abuse. CG did not have any psychiatric or physical disorders.
								8 (CG) Males					
[166]	Wasti et al.	2013	Pakistan	120	44	29.07 ± 9.05 (SG treated with haloperidol) 27.0 ± 7.10 (SG treated with clozapine)	ND	ND	Fe	ND	SG presented decreased serum Fe and ferritin levels, and increased level of total iron-binding capacity (TIBC) in SG treated with haloperidol compared to CG. Patients treated with clozapine did not show any significant changes in serum Fe levels, except for lowered ferritin levels.	SG was on a chronic haloperidol (*n* = 92) and clozapine (*n* = 28) treatment for more than 12 weeks.	CG included only patients with o history of psychiatric conditions, or other concurrent diseases. Besides serum Fe levels, TIBC, and serum ferritin levels were measured.
[167]	Barnes et al.	1992	UK	105	ND	31–65 (range)	ND	26 (SG) Females	Fe	ND	There was no significant correlation between serum Fe levels and daily dose of antipsychotic drug (r = 0.03). There was also a significant correlation between serum Fe levels in groups either receiving and those who were not receiving oral antipsychotic medication. There was no strong relationship between serum Fe concentrations and the severity of akathisia(r = 0.16, *p* = 0.45). Serum Fe levels were lower in chronic akathisia patients compared to those without akathisia, however, without a statistical significance (*p* = 0.224). Serum Fe levels were higher in the pseudoakathisia group compared to non-akathisia group, and significantly higher compared to chronic akathisia group (*p* = 0.051 and *p* = 0.004 respectively). Serum Fe levels were slightly higher in males compared to females, but without any statistical significance.	For each patient, the antipsychotic drug was converted to chlorpromazine equivalents in mg per day. 26 patients were taking depot, but not oral medication, whereas 3 patients were not receiving any antipsychotic drugs. Patients with chronic akathisia were taking higher doses of antipsychotic drugs compared to those without akathisia.	A total sample of 105 subjects included: 24 with chronic akathisia, 21 with pseudoakathisia, and 60 with no akathisia. The severity of akathisia was mild in 13 cases, moderate in 8, marked in 2, and severe in 1 case.
								79 (SG) Males					
[168]	Hofmann et al.	2000	Switzerland	33	23	38.5 ± 14.5	34.0 ± 9.6	12 (SG) Males 21 (SG) Females	Fe	ND	SG has serum Fe levels, which were within physiological range. Male patients had slightly higher serum Fe levels than females, and pre-menopausal females had higher serum Fe levels than post-menopausal serum Fe levels. However the above-mentioned results were not statistically significant. No significant differences were found between SG and CG.	Neuroleptic medicamention used in either akathisic or non-akathisic group included: haloperidol, benperidol, bromperidol, perazine, flupenthixol, trifuopenazine, pimozide, chlopenthixol, or chlorpromazine. None of the patients received atypical antipsychotics.	SG included 33 subjects among whom 18 had SZ—13 paranoid, episodic, and 5 disorganized, catatonic type. Non-akathisic group included 13 SZ patients—8 paranoid, episodic, and 5 disorganized, catatonic. SG (akathisia group), despite SZ, also included patients with schizoaffective disorder, affective disorder (psychotic mania or depression), or drug-induced psychosis (amphetamine). CG included patients with SZ, schizoaffective disorder, or mania with psychotic features. Besides serum Fe, serum ferritin was studied in both groups.
								13 (CG) Males 10 (CG) Females					
[169]	Kim et al.	2018	Korea	121	ND	22.0–32.0 (range)	ND	73 (SG) Females	Fe	ND	Latent Fe deficiency is significantly associated with negative symptoms of SZ. SZ patients with prominent negative symptoms have also significantly lower ferritin levels.	Patients were taking: amispulrpide (*n* = 32), aripiprazole (*n* = 21), paliperidone (*n* = 53), risperidone (*n* = 5), quetiapine (*n* = 7), and none (*n* = 3).	Inclusion criteria were duration of treatment for psychotic symptoms less than 2 years, first episode of schizophrenia, schizophreniform disorder, or other specified schizophrenia spectrum disorder and duration of treatment less than 2 months. Exclusion criteria included: age less than 18 years, diagnosis of a substance or medication induced psychotic disorder, psychotic disorder due to other medical conditions, or other concurrent severe medical conditions. Among 121 subjects, 77 were diagnosed with SZ, 32 were schizophreniform, 12 were other specified.
								48 (SG) Males					
[170]	Kuloglu et al.	2003	Turkey	60	30	34.8 ± 9.5	35.1 ± 9.2	14 Females and 16 Males (SG with akathisia)	Fe	Serum Fe was measured using Olympus AU600 autoanalyzer.	Serum Fe levels were significantly lower in the akathisic group (*p* < 0.001) and non-akathisic groups (*p* < 0.001) compared to CG. TIBC was in akathisic patients compared to CG (*p* < 0.01). Ferritin levels decreased in both SG groups compared to CG (*p* < 0.01). Transferrin levels were not statistically different among studied groups.	SZ patients were treated with chlorpromazine.	SG included patients with SZ—30 with akathisia, and 30 non-akathistic. Other studied parameters included TIBC, ferritin, and transferrin.
								14 females and 16 males (SG without akathisia)					
								15 females and 15 males (CG)					
[171]	Soni et al.	1993	UK	22	22	52	ND	9 (SG) Males	Fe	Serum Fe levels were measured by the ferrozine method.	SG presented a higher level of psychopathology and positive symptoms; such differences were not observed in case of negative symptoms. Serum Fe, iron binding capacity, and ferritin levels did not differ significantly between both groups. Only 2 patients showed low serum Fe concentrations, but finding was not associated with increased TIBC. Serum ferritin levels were low in 14 patients—6 from SG, and 8 from CG. 1 patient from each group had increased TIBC with low serum ferritin levels. There was no correlation between akathisia severity and serum Fe levels (*p* = 0.36), serum iron binding capacity (*p* = 0.11), or serum ferritin (*p* = 0.49).	All patients except for four subjects were receiving depot neuroleptic drugs, either alone (11 subjects), or in combination with oral neuroleptics. 15 patients (8 from akathisia group), received anticholinergic drugs (+ neuroleptic medication) a fortnight before testing. 3 subjects were treated with antidepressants.	Patients who were included in the study had to be in good physical health and on no other medication than neuroleptics. Exclusion criteria were organic brain syndromes, a history of alcoholism or drug abuse, Fe therapy, cimetidine, or antacids. A mean duration of illness was estimated to 22.8 ± 14.9.
								13 (SG) Females					
[172]	Spina et al.	1994	Italy	17 ***	16	42.5 ± 5.3	43.1 ± 4.8	4 (SG) Females 13 (SG) Males	Fe	Serum Fe concentrations were measured using a colorimetric method.	There were no significant differences in Fe, ferritin, and transferrin levels in both groups.	Patients were chronically treated with oral neuroleptics and antiparkinsonian drugs. Later, neuroleptic drugs were converted to chlorpromazine equivalents.	SG constituted a group of 17 subjects who have experienced one or more dystonic reactions during neurolpetic treatment. A CG constituted of 16 SZ, however, without a history of neuroleptic-induced extrapyramidal disorders. Exclusion criteria were non-steroid anti-inflammatory drugs, H_2_-histamine antagonists, or antacids.
								4 (CG) Females 12 (CG) Males					
				44 ****	ND	19–39 (range)	ND	44 (SG) Males	Fe		Serum Fe levels did not differ significantly between patients with or without dystonia, either on admission, or after 3 weeks before neuroleptic treatment.	37 patients were treated with haloperidol, 7 with bromperidol.	6 patients experienced acute dystonic reactions during the observation period.
[173]	Wirshing et al.	1998	USA	30	ND	40.1 ± 8.2	ND	30 (SG) Males	Fe	ND	There is a significant correlation between score of AIMS and serum ferritin levels (*p* = 0.018). There was no significant correlation between AIMS score and serum Fe and TIBC capacity levels. There was no significant correlation between serum Fe levels and akathisia ratings or choreoathetoid movement ratings. Only serum ferritin levels are associated significantly with choreoathetoid movements in male SZ patients chronically treated with fluphenazine decanoate.	All of the subjects from SG were treated with fluphenazine decanoate every two weeks. The mean duration of treatment was 73 ± 51 weeks.	The severity of choreoathetoid movements was assessed with AIMS and akathisia was assessed using Barnes scale.
[174]	Weiser et al.	1994	Israel	26	12	16–76 (range)	24–51 (range)	16 (SG) Males 10 (SG) Females	Fe	Fe concentration was determined using a Krone diagnostic kit.	Mean morning serum Fe levels were lower in SG compared to CG, however, the difference was not statistically significant (*p* = 0.17).	Patients were medication free for minimum one month before admission.	SG consisted of subjects with either SZ or schizoaffective disorder.
								11 (CG) Males 1 (CG) Female					
[175]	Alexander et al.	1978	USA	31	173	24 ± 1	22 ± 1	16 (SG) Males	Ca, Mg	Ca and Mg levels were determined by atomic absorption spectroscopy.	SZ patients did not show any statistical difference in serum Ca and Mg levels compared to CG. Significantly lower serum Ca levels were observed in patients who remitted a following neuroleptic withdrawal compared to patients who relapsed (*p* < 0.02). SZ patients treated with pimozide showed decreased serum Mg and Ca levels. Generally, SZ patients present with physiological ranges of serum Ca and Mg, which are decreased only during neuroleptic treatment.	Pimozide, fluphenazine (either hydrochloride or decanoate), benztropine, diphenhydramine (the last two were prescribed when extrapyramidal symptoms occurred)	SG consisted of subjects with either SZ od schizoaffective disorder. Blood tests performed on admission show physiological serum concentrations of Ca, Mg, Na, K, P, and Cl.
								15 (SG) Females					
[176]	Alexander et al.	1979	USA	22	ND	22	ND	11 (SG) Females	Ca, Mg	Ca and Mg levels were determined by atomic absorption spectroscopy.	During pimozide treatment, among 22 SZ patients, 16 developed extrapyramidal symptoms. These 16 patients with EPS had significantly lower drug-free serum Ca levels compared to patients without EPS (*p* < 0.05). Serum drug-free Ca or Mg levels did not correlate with the dosage of pimizode at which EPS started. However, serum Ca and Mg levels together correlated with pimizode dosage at which EPS developed. Higher drug-free serum Ca and Mg levels were observed in patients who tolerated higher doses of neuroleptics.	Pimozide treatment (1–2 mg per day) until extrapyramidal symptoms developed or maximal clinical improvement was reached. Benztropine or diphenhydramine were prescribed were needed during neuroleptic treatment trials.	SG included patients diagnosed with SZ or schizoaffective disorder. Blood tests performed on admission show physiological serum concentrations of Ca, Mg, Na, K, P, and Cl.
								11 (SG) Males					
[177]	Athanassenas et al.	1983	Greece	29	ND	46.3 ± 1.8		14 (SG) Males	Ca, Mg	Ca and Mg levels were determined by atomic absorption spectroscopy.	During drug-free period, serum Ca and Mg levels did not significantly differ compared to CG. No significant differences were stated between females and males. Both serum Ca and Mg decreased significantly during treatment (*p* < 0.001). 19 patients, who developed EPS, had nearly identical serum Ca and Mg levels compared to CG, whereas 10 patients without EPS had lower drug-free serum Ca levels and higher serum Mg levels. 3 male patients developed catatonic episodes, and at the beginning of these episodes presented significantly higher Ca levels (*p* < 0.02), whereas Mg increased, but without statistical significance.	Patients were treated with pimozide (*n* = 9), fluphenazine (*n* = 8), and loxapine (12). Trihexyphenidyl or biperiden were prescribed when EPS occurred.	The mean hospitalization duration was 9.6 ± 1 year. Among 15 female patients, 8 were postmenopausal. All of the patients did not have any additional concurrent medical conditions. Patients were drug free 3 weeks before blood collection.
								15 (SG) Females					
[178]	Levine et al.	1994	Israel	16	ND	42.7 ± 24 (acute) 48.3 ± 19 (remission)	ND	ND	Ca, Mg	Ca and Mg levels were determined by the colorimetric determination.	There was no correlation between dosage of neuroleptics and serum Ca and Mg concentrations.	Treatment included neuroleptic drugs (chlorpromazine equivalents)—820 mg/day in acute and 470 mg/day in remission schizophrenics.	Among SG, 8 SZ patients were acute and 8 were on remission. All of the patients did not have any concurrent disease. Despite serum Ca and Mg levels, also Ca and Mg levels were determined in cerebrospinal fluid.
[179]	Alertsen et al.	1986	Norway	61	35	ND	ND	17 (CG) Males	Se	Se levels were determined by fluorimetric method.	There was no statistical difference in serum Se concentrations between SG and CG.	ND	A group of 61 psychiatric patients included: 17 schizophrenics, 7 with paranoid disorder, 6 with affective psychosis, 4 with reactive psychosis, 11 with dementia senilis, 17 with neurosis and psychopathia, and 2 with delirium tremens.
								18 (CG) Females					
[180]	Ruljancic et al.	2013	Croatia	71	99	45 ± 12.9 (a group without suicide attempt) 42 ± 15.6 (a group who attempted suicide)	49 ± 11.6	28 (SG without suicide attempt) Males 20 (SG without suicide attempt) Females	Ca, Mg	Serum Ca and Mg levels were measured using atomic absorption spectrophotometry.	Serum Ca levels were lower in SG with a medical history of suicide attempt compared to both CG and SG without suicide attempt. There were no differences in serum Mg levels between the three groups.	ND	Among 71 subjects from SG, 48 patients were schizophrenics without history of suicide attempt, whereas 23 patients had attempted suicide.
								7 (SG who attempted suicide) Males 16 (SG who attempted suicide) Females					
								48 (CG) Males 51 (CG) Females					
[181]	Jamilian et al.	2012	Iran	68 (SZ group) 35 (depression group)	50	35.67 ± 10.46 (SZ group) 35.84 ± 8.20 (depression group)	35.26 ± 4.82	68 (SG SZ group) Males 35 (SG depression group) Males	Ca, P	Serum Ca and P levels measured using routine laboratory methods.	Serum P levels were significantly lower in schizophrenics, compared to healthy controls (*p* = 0.016); there was no difference in serum P levels between depressed patients and either schizophrenics (*p* = 0.379) or healthy subjects (*p* = 0.323). Final results showed that only vitamin D (and not Ca, P, or parathyroid hormone) levels are lower in psychiatric patients compared to CG.	ND	Exclusion criteria were: renal insufficiency, liver dysfunction, parathyroid disorders, administration of drugs which might alter Ca, P, vitamin D, or parathyroid hormone levels. All of the patients were in an acute stage of the disease. 7 schizophrenic patients suffered from following diseases: 2 were diabetics, 2 had hyperlipidemia, 1 had thyroid dysfunction, 1 had a history of seizure attacks, and 1 had a history of head trauma. 12 schizophrenic patients were abusing drugs: 8 used opium, 2 cannabis, crack, and 1 multiple drugs. 11 schizophrenic patients had a positive family history.
								50 (CG) Males					
[182]	Lin et al.	2017	China	114	114	32.8 ± 11.3	32.9 ± 10.7	ND	Al, As, B, Ba, Bi, Ca, Cd, Ce, Co, Cr, Cs, Cu, Fe, Gd, Ge, Hg, La, Li, Mg, Mn, Mo, Na, Nd, Ni, P, Pb, Pr, Rb, S, Sb, Se, Sn, Sr, Th, Ti, Tl, U, V, Zn	Serum elements levels were measured by ICP-MS and inductively coupled plasma atomic emission spectroscopy (ICP-AES).	Serum Al, As, B, Bi, Ca, Cd, Co, Cu, Fe, Gd, Li, Mn, Na, Ni, P, Rb, S, Se, Sn, Sr, Th, Ti, Tl, and V differed significantly between schizophrenics group and control group (all *p* < 0.05).	ND	Inclusion criteria were: age between 18 and 65, no exposure to heavy industry, no additional diseases (infectious, traumatic), no use of hormone therapy. Patients who were taking mineral or vitamin supplements were excluded.
[183]	Cai et al.	2015	China	50 (SZ) * 49 (CG) *	61 (SZ) ** 61 (CG) **	36.8 ± 2.5 (SZ) * 28.4 ± 8.5 (CG) *	36.9 ± 2.02 (SZ) ** 36.9 ± 9.7 (CG) **	21 (SZ) * Males 29 (SZ) * Females	Ag, Al, As, B, Ba, Be, Bi, Ca, Cd, Co, Cr, Cs, Cu, Er, Fe, Ga, Ge, Li, Mg, Mn, Mo, P, Pb, Rb, Sb, Se, Sr, Tb, Te, Ti, Tl, U, V, Yb, Zn	Serum elements levels were determined by the ICP-MS.	Only serum levels of Cs, Zn, and Se were significantly reduced in SZ patients compared to healthy controls in both the training group (*p* = 0.0004, 0.0002, and 0.0004 respectively) and the test group (*p* = 1.4 × 10^−6^, 2.8 × 10^−6^ and 7.3 × 10^−6^ respectively). For all samples, serum P, Pb, and Yb levels were significantly associated with schizophrenia.	Serum was collected before the initiation of antipsychotic treatment.	Inclusion criteria were: no cases of alimentary restriction or clinical malnutrition, no history of substance abuse, no current drug or supplement intake for at least one month, no concurrent disorders which are known to affect trace element metabolism and levels.
								1 (CG) * Males 48 (CG) * Females					
								25 (SZ) ** Males 36 (SZ) ** Females					
								25 (CG) ** Males 36 (CG) ** Females					
[184]	Ma et al.	2018	China	109	106	18–65 (range)	18–65 (range)	47 (SG) Males 62 (SG) Females	Pb, Mg, Cr, Sb, Ag, U	Elements concentrations were measured using ICP-MS.	Only Ag, Sb, and U levels had 100% detection rates in sera of the studied patients. Sb and U levels were significantly higher in both case and control groups. Sb and U levels did not differ significantly between first-episode and recurrent SZ patients. Higher Sb and U serum concentrations are associated with the higher risk of schizophrenia.	Patients did not take any antipsychotic drugs for at least 1 month before hospitalization.	Inclusion criteria were: 18–65 years of age, no previous exposure to heavy industry, no acute infections and traumatic diseases, first-episode and drug-naive schizophrenia, no use of hormone therapy. Exclusion criteria were: concurrent psychiatric disorders, additional metabolic or chronic diseases, receiving mineral or vitamin supplements.
								38 (CG) Males 68 (CG) Females					
[185]	Ma et al.	2019	China	95	95	18–60 (range)	18–60 (range)	41 (SG) Males 54 (SG) Females	Cr, Cd, Pb, As	Elements concentrations were measured using ICP-MS.	Serum concentration of As was significantly lower in SG compared to CG (*p* < 0.05) while serum Pb level was significantly higher in SG compared to CG. Serum Pb levels, under drug-free conditions are associated with an increased risk of schizophrenia.	ND	The inclusion criteria were: 18–60 years of age, no history of exposure to heavy industry, no acute infectious and traumatic diseases, no history of chronic physical illness, not receiving mineral or vitamin supplements.
								49 (CG) Males 46 (CG) Females					

ND—no data, SG—studied group, CG—control group, SZ—schizophrenia, PANSS—positive and negative syndrome scale, AIMS—abnormal involuntary movement scale, EPS—extrapyramidal symptoms, *—test group, **—training group, ***—cross-sectional study, ****—prospective study, ^1^ Collectively—studied and control group.

## Abbreviations

AChE	acetylcholinesterase
ADHD	attention deficit hyperactivity disorder
Al	aluminum
ALT	alanine aminotransferase
ASD	autism spectrum disorder
AST	aspartate transaminase
Ca	calcium
Cd	cadmium
CNS	central nervous system
Cu	copper
Fe	iron
GABA	γ-aminobutyric acid
GABAA	γ-aminobutyric acid type A
Gln	glutamine
Glu	glutamate
GPx	glutathione peroxidase
HDL	high-density lipoprotein
hs-CRP	high-sensitivity C-reactive protein
IDD	iodothyronine deiodinases
K	potassium
LDL	low-density lipoprotein
Li	lithium
Mg	magnesium
Mn	manganese
MnSOD	manganese superoxide dismutase
Mo	molybdenum
Ni	nickel
NMDA	N-methyl-D-aspartate
P	phosphorus
PMCA	plasma membrane Ca^2+^-ATPase
Rb	rubidium
Sb	antimony
Se	selenium
SELENBP1	selenium binding protein 1 gene
SelH	selenoprotein H
SelP	selenoprotein P
SelS	selenoprotein S
TIBC	total iron-binding capacity
TrxR	thioredoxin reductase
U	uranium
VLDL	very-low-density lipoprotein
Zn	zinc
